# Urgent Myectomy for Hemodynamic Instability: Leveraging the Advantages of a Perceval Sutureless Valve for Unexpected Systolic Anterior Motion After Declamping the Aorta

**DOI:** 10.7759/cureus.80944

**Published:** 2025-03-21

**Authors:** Yoshun Sai, Kunihiko Yoshino, Joji Ito

**Affiliations:** 1 Cardiovascular Surgery, Tokyo Bay Urayasu Ichikawa Medical Center, Chiba, JPN

**Keywords:** adult cardiac surgery, mitral valve plasty, myectomy, sutureless aortic valve replacement (suavr), systolic anterior motion of the mitral valve

## Abstract

Sutureless aortic valve replacement (AVR) has been widely recognized for its ability to reduce aortic cross-clamp and cardiopulmonary bypass times while allowing for intraoperative repositioning or reimplantation. However, unexpected complications such as systolic anterior motion (SAM) can arise, necessitating additional surgical interventions.

We report the case of a 61-year-old male with a history of hypertension and Behçet’s disease in his youth, which had remained clinically inactive. He presented with worsening dyspnea, and preoperative echocardiography revealed moderate aortic stenosis with a bicuspid aortic valve and moderate mitral regurgitation secondary to chordal rupture. Although the patient did not strictly meet the criteria for SAM risk, he had a mildly hypertrophied interventricular septum measuring 13 mm. The patient underwent concomitant mitral valve repair and sutureless AVR using a Perceval valve. Following aortic declamping, intraoperative transesophageal echocardiography revealed severe SAM and left ventricular outflow tract obstruction with worsening mitral regurgitation. Notably, septal hypertrophy was more pronounced intraoperatively, and the left ventricular morphology was determined to be a significant contributing factor to SAM. Given the hemodynamic instability, a myectomy was performed through the aortic valve approach. The sutureless Perceval valve was easily removed and reimplanted, allowing for rapid completion of the procedure without excessive prolongation of myocardial ischemia. Compared to a standard bioprosthesis, the ease of valve removal and repositioning provided a crucial advantage in this setting, facilitating prompt surgical intervention.

Postoperatively, the patient recovered well, with no residual SAM or mitral regurgitation on follow-up echocardiography.

This case highlights the utility of sutureless AVR in complex cardiac surgery, particularly in scenarios requiring additional intraoperative interventions. The ability to promptly remove and reposition the valve enabled effective management of SAM while minimizing ischemic time, underscoring its advantage over conventional bioprosthetic valves in such situations.

## Introduction

Sutureless aortic valve replacement (AVR) has garnered increasing attention in recent years for its potential to decrease aortic cross-clamp and cardiopulmonary bypass times while enabling straightforward intraoperative repositioning or reimplantation of the prosthesis [1]. This technique has been increasingly adopted due to its advantages in both routine and complex cases, including multi-valve procedures [2]. This advantage is particularly valuable in complex, multi-valve procedures where operative flexibility can significantly influence outcomes, especially in cases requiring intraoperative valve adjustments due to malpositioning or complications such as systolic anterior motion (SAM).

SAM is a phenomenon in which the anterior mitral valve leaflet or interventricular septum obstructs the left ventricular outflow tract (LVOT) during systole, often leading to dynamic LVOT obstruction and hemodynamic instability. It is commonly associated with hypertrophic cardiomyopathy but can also occur due to factors such as hyperdynamic left ventricular function, an elongated mitral valve, or changes in ventricular geometry following cardiac surgery [3,4].

Here, we present a case of unexpected SAM following aortic declamping, prompting an additional myectomy. Preoperatively, the patient exhibited a mildly hypertrophied interventricular septum (13 mm), which, while not strictly meeting the criteria for SAM risk, may have contributed to its development. By capitalizing on the rapid removal and redeployment features of a Perceval sutureless valve, it was possible to carry out the extra procedure without substantially prolonging myocardial ischemia time. In comparison to conventional AVR using a standard bioprosthesis, where valve removal and reinsertion could be more time-consuming and technically challenging, the sutureless valve facilitated a more efficient intervention in this case. Notably, this additional procedure resulted in only 49 minutes of additional ischemic time, demonstrating the practical advantage of this approach.

## Case presentation

The patient was a 61-year-old man who presented with dyspnea. He had a history of hypertension and Behçet’s disease. Three days prior to admission, he visited a local clinic with fever and dyspnea, was prescribed oral antibiotics and antipyretic/analgesic agents, and was advised to continue observation. One day before admission, he returned with worsening dyspnea; acute mitral regurgitation (MR) leading to heart failure was diagnosed, and he was admitted to the referring hospital. He was transferred to our institution the following day for further evaluation and treatment.

**Figure 1:**
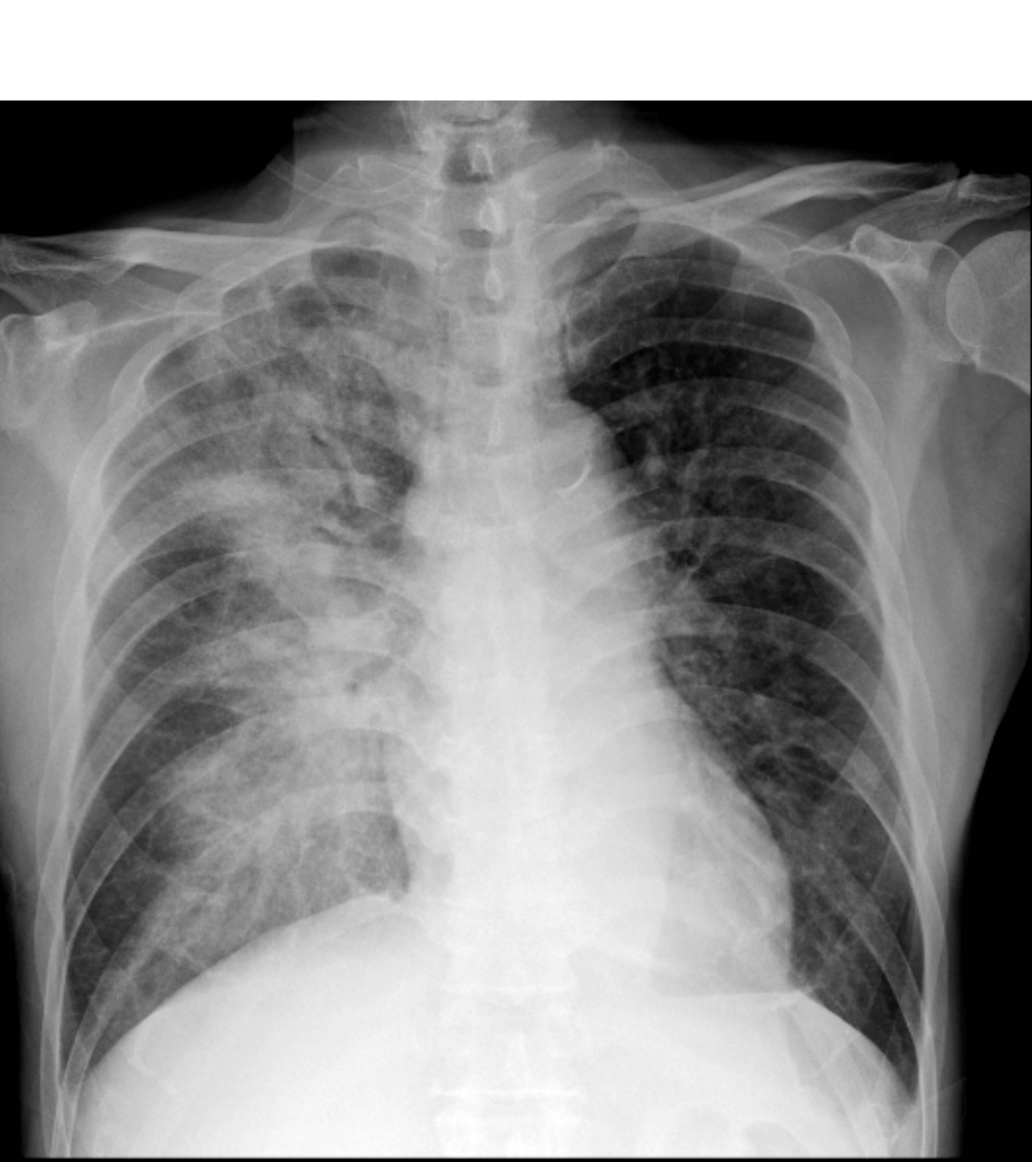
Chest X-ray The chest X-ray shows ground-glass opacities in the right lung field, suggesting pulmonary congestion secondary to acute mitral regurgitation.

On admission, he was 160 cm tall, weighed 58 kg, and had a blood pressure of 120/70 mmHg, heart rate of 90 bpm, and temperature of 38.0°C. A Levine III/VI systolic murmur was auscultated best at the left sternal border in the third intercostal space; there was no jugular venous distension or bilateral lower-limb edema. Laboratory findings revealed the following: white blood cell count 14,500/μL, hemoglobin 14.7 g/dL, platelets 194,000/μL, blood urea nitrogen 18.9 mg/dL, creatinine 0.71 mg/dL, aspartate transaminase 20 IU/L, alanine transaminase 22 IU/L, and NT-proBNP (N-terminal prohormone of brain natriuretic peptide) 173.2 pg/mL. The chest X-ray showed a cardiothoracic ratio of 45% and ground-glass opacities in the right lung field, suggesting pulmonary congestion secondary to acute MR (Figure 1).

Electrocardiography indicated sinus rhythm at 95 bpm and ST depression in leads II, III, and aVF. Transthoracic echocardiography showed a left atrial diameter of 39 mm, septal/posterior wall thickness of 13/11 mm, left ventricular end-diastolic diameter/left ventricular end-systolic diameter of 48/21 mm, and a left ventricular ejection fraction of 64% with normal wall motion, indicating a mildly hypertrophied interventricular septum. The aortic valve exhibited a peak velocity of 2.7 m/s, mean pressure gradient of 15 mmHg, and a valve area of 1.04 cm², with mild regurgitation. Moderate MR was noted due to chordal rupture of P3 (regurgitant volume 36 mL, regurgitant fraction 40%, effective regurgitant orifice 0.20 cm²). Transesophageal echocardiography demonstrated chordal rupture of P3, leading to regurgitation in P3 (Video 1), as well as a bicuspid aortic valve (fusion of the right and left coronary cusps) with moderate aortic stenosis (valve area 0.84 cm²).

**Video 1:** Transesophageal echocardiography showing chordal rupture of P3.

We diagnosed the patient with acute MR and moderate aortic stenosis and decided to proceed with an urgent surgical intervention.

**Figure 2:**
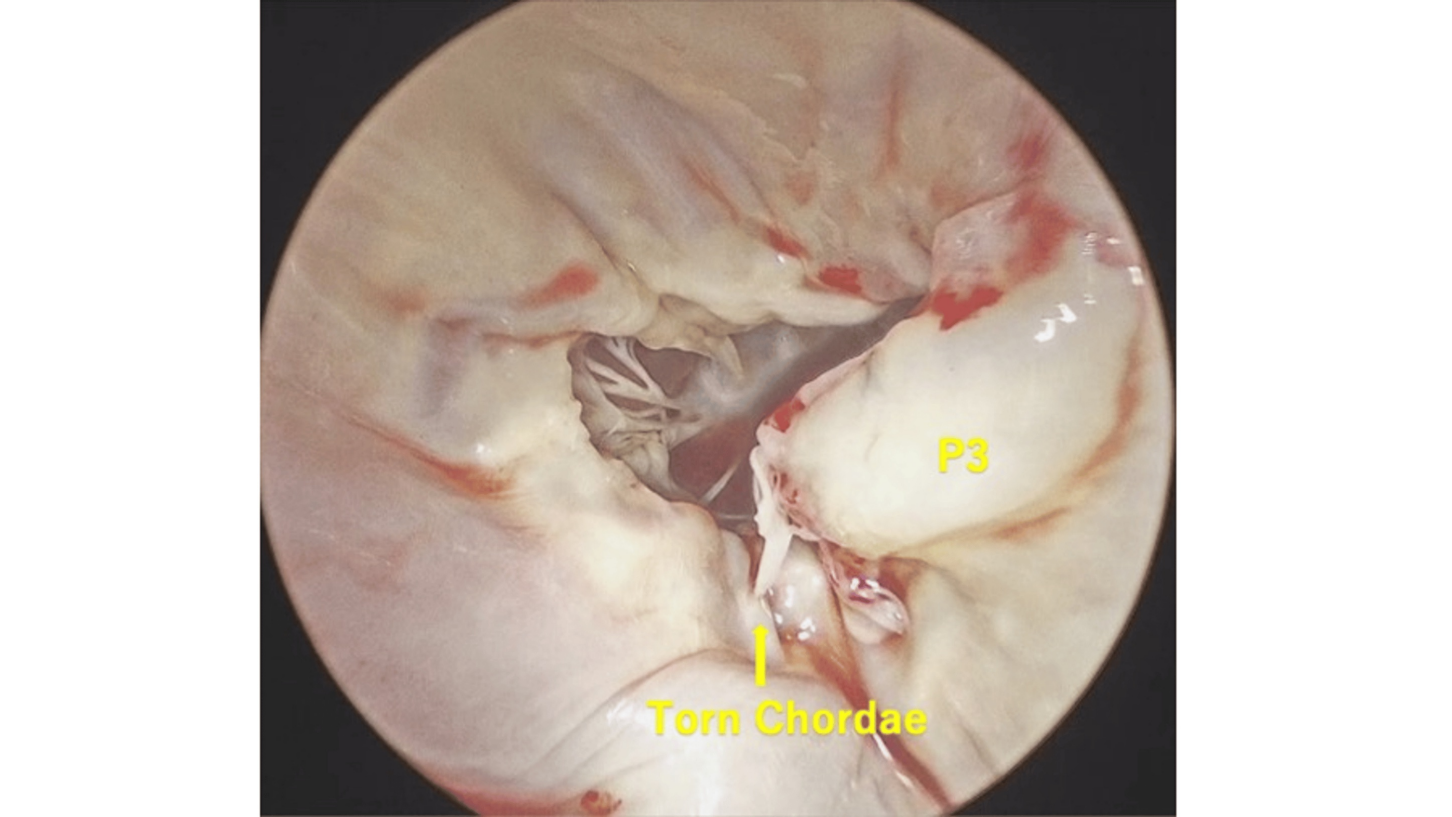
Intraoperative findings revealed chordal rupture and prolapse of the P3 segment (yellow arrowheads).

Under general anesthesia, a median sternotomy was performed. Cardiopulmonary bypass was established with inflow via the ascending aorta and venous drainage from the superior and inferior vena cavae. A left atrial vent was placed through the right superior pulmonary vein, and a root cannula was inserted in the ascending aorta. After clamping the aorta, an antegrade cardioplegic solution was administered to achieve cardiac arrest. A superior transseptal approach allowed visualization of the mitral valve, revealing a wide prolapse of the P3 segment (Figure 2).

Two pairs of artificial chordae (CV-4) were anchored to the posterior papillary muscle, with each pair looped twice around the medial and lateral edges of the prolapsed P3 segment. A 29-mm Tailor Annuloplasty Band (St. Jude Medical, Saint Paul, MN, USA) was used for annuloplasty, adjusting chordal length through water testing. Because the P3 segment measured only 10 mm, leaving no additional resection margin, artificial chordae were selected. After closing the atrium, the ascending aorta was opened. The aortic valve was confirmed to be bicuspid (fusion of the right and left cusps), and thus the cusps were excised and the annulus decalcified. Three equally spaced points on the annulus were marked, and 4-0 polypropylene guiding sutures were placed. A Perceval sutureless valve (Corcym, Saluggia, Italy) size L was deployed via the tourniquet technique and then repositioned around the membranous septum and balloon-dilated at 4 atm for 30 seconds. After declamping the aorta, transesophageal echocardiography revealed severe SAM and MR, apparently due to pronounced hypertrophy of the LVOT (Video 2). A decrease in left ventricular afterload resulting from the resolution of aortic stenosis, coupled with anatomical modifications caused by mitral valve repair, likely played a role in the onset of SAM by exacerbating LVOT obstruction.

**Video 2:** Transesophageal echocardiography showing severe SAM and MR due to pronounced LVOT hypertrophy. Transesophageal echocardiography revealed severe SAM and MR, apparently due to pronounced hypertrophy of the LVOT, which remained unresponsive to fluid administration and β-blocker therapy. SAM, systolic anterior motion; MR, mitral regurgitation; LVOT, left ventricular outflow tract

**Figure 3:**
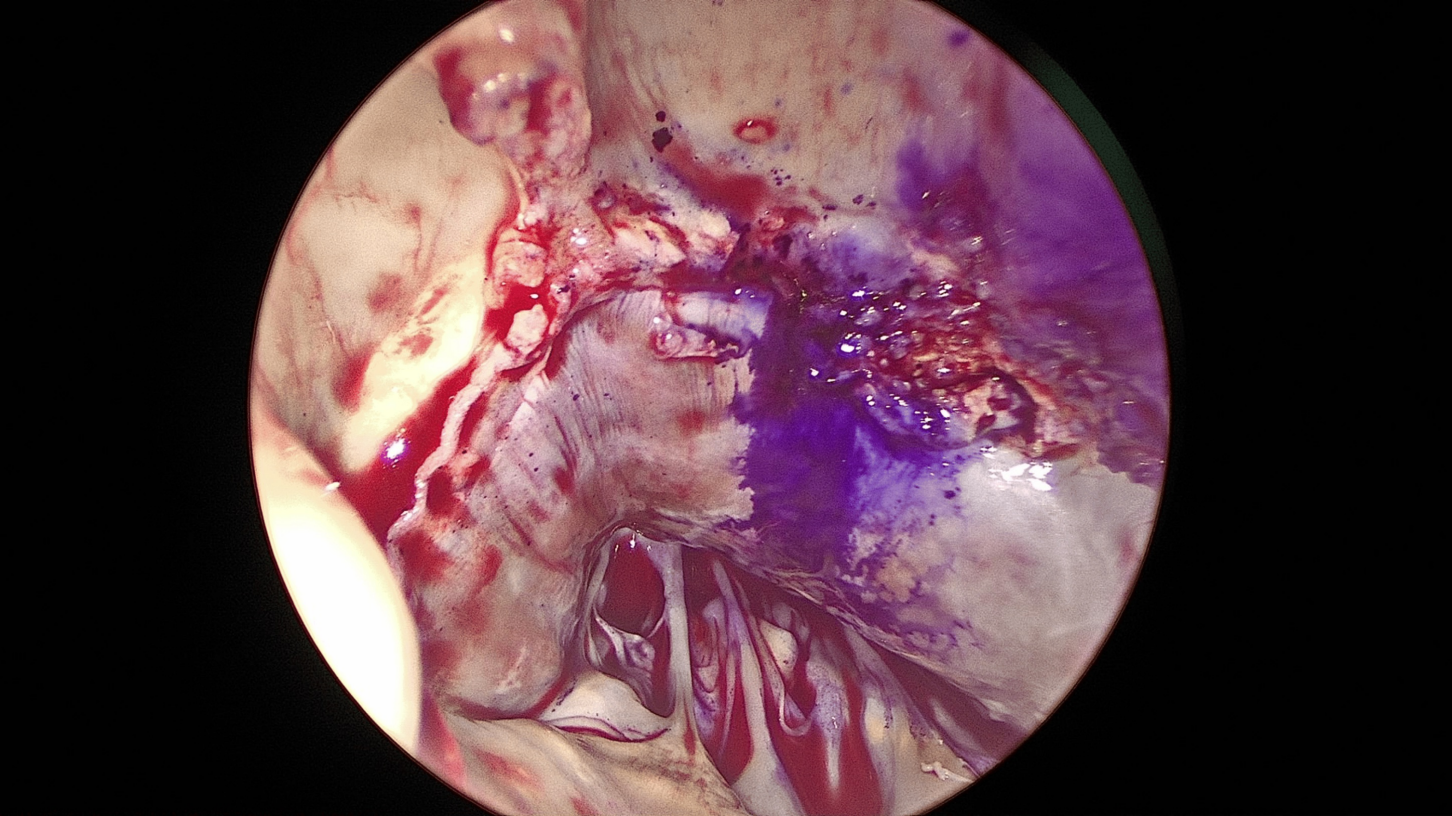
Myocardial extension into the LVOT observed through the aortic valve. A marker was placed at the nadir of the left coronary cusp to avoid damage to the conduction system. LVOT, left ventricular outflow tract

**Figure 4:**
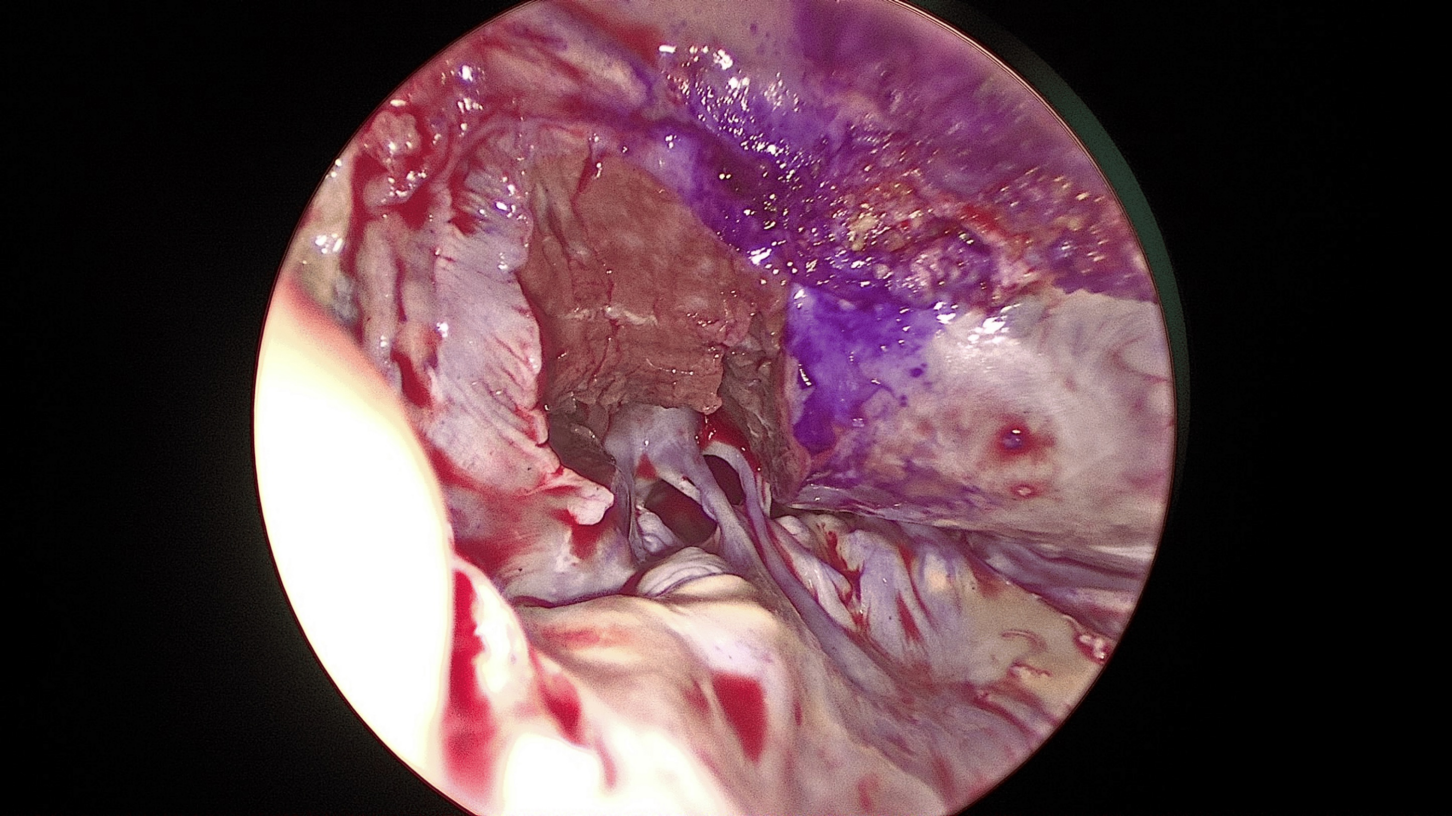
Extended myocardial resection from the right coronary cusp to the nadir of the left coronary cusp Myocardial resection was performed from the right coronary cusp to the nadir of the left coronary cusp, extending toward the apex beyond the level of the papillary muscles.

The aorta was reclamped and reopened to perform a myectomy, with easy removal of the Perceval valve allowing direct access through the aortic valve. The hypertrophic myocardium beneath the right and left coronary cusps, extending toward the apex and beyond the papillary muscles, was resected (Figures 3, 4).

The Perceval valve was then reimplanted in the same manner as before. After declamping the aorta, transesophageal echocardiography confirmed that SAM had resolved (Video 3). The second cross-clamp time required for removing the Perceval valve and performing the myectomy was 49 minutes.

**Video 3:** Transesophageal echocardiography after myectomy. Subsequent to the myectomy, the previously observed systolic anterior motion was definitively resolved.

Postoperatively, the patient was extubated for 15 hours after surgery. It was found that the blood cultures obtained at the time of admission were negative. On postoperative day 5, transthoracic echocardiography showed no MR or SAM, and the mean mitral valve pressure gradient was 4 mmHg. He made an uneventful recovery and was discharged home under his own ambulation on postoperative day 8. Six months later, he remains in NYHA functional class I and continues outpatient follow-up. Given the presence of a prosthetic aortic valve and prior hypertrophy, future LVOT gradient progression remains a concern. Serial echocardiography is planned to monitor for potential LVOT obstruction or valve dysfunction.

## Discussion

Sutureless AVR has been shown in a systematic review and meta-analysis to shorten aortic cross-clamp and cardiopulmonary bypass times, thereby reducing overall operative risk [1]. The ability to rapidly implant or reposition the prosthesis becomes especially important when unexpected intraoperative complications, such as SAM, demand prompt intervention to maintain hemodynamic stability.

The advantages of sutureless valves are further demonstrated in complex surgical settings involving multiple or concomitant procedures. Baran et al. [2] reported that patients who underwent sutureless AVR in conjunction with additional valvular surgeries experienced favorable operative outcomes, emphasizing how a rapid-deployment prosthesis can offer flexibility and efficiency. Although their study did not center on acute complications such as SAM, the principle of minimizing ischemic time and simplifying implantation remains essential when faced with sudden, urgent challenges.

SAM itself is a well-recognized complication after mitral valve repair, arising when the anterior leaflet is pulled into the LVOT during systole, obstructing ejection or causing severe MR. First described in the 1970s [3,4], its documented risk factors include left ventricular end-systolic diameter (LVESD) < 35 mm, left ventricular end-diastolic diameter (LVEDD) < 45 mm, a leaflet-to-leaflet distance < 25 mm, a posterior leaflet height > 15 mm, and a basal septal thickness > 15 mm [5,6].

Management strategies for SAM include conservative measures such as β-blockers to reduce heart rate and myocardial contractility, fluid administration to enhance preload, and vasoconstrictors to increase afterload. However, if these medical interventions prove ineffective or if hemodynamic instability ensues, reinduction of cardiac arrest and surgical intervention become necessary. Surgical approaches include shortening artificial chordae on the posterior leaflet, excising redundant posterior leaflet tissue, modifying the annuloplasty ring size, or changing the ring type (e.g., from a flexible ring to a rigid ring). However, in cases in which SAM is exacerbated by LVOT obstruction, mitral valve reconstruction alone may be insufficient for adequate management.

In the present case, preoperative echocardiography did not reveal all the conventional risk factors for SAM. Nevertheless, a combination of reduced left ventricular afterload following the resolution of aortic stenosis and the anatomical changes induced by mitral valve repair likely contributed to the development of SAM by worsening LVOT obstruction. Various management strategies were considered, including the addition of an Alfieri stitch, conversion to a rigid ring, further resection of the posterior leaflet, removal of the annuloplasty ring, and myectomy. The Alfieri stitch was deemed unsuitable due to the high risk of inducing mitral stenosis. Conversion to a rigid ring was also considered inappropriate, as the anteroposterior diameter of the annulus was narrow (26 mm), and the A2 segment was relatively short (23 mm), raising concerns about inadequate coaptation. Furthermore, the P3 segment was only 10 mm in length, leaving no viable margin for additional resection. Intraoperative transesophageal echocardiography confirmed these anatomical limitations, reinforcing the decision to avoid excessive resection or annuloplasty modifications. After evaluating the options of annuloplasty ring removal or myectomy, we capitalized on the easy explantation and redeployment capability of the Perceval valve, allowing us to perform myectomy through the aortic valve approach efficiently.

One recognized drawback of sutureless valves is their potential to cause conduction disturbances, with a 4.4% permanent pacemaker implantation rate documented in Japan [7]. Compared to conventional sutured bioprostheses, which exhibit pacemaker implantation rates ranging from 3.5% to 8.0% in similar settings [8,9], this rate is within an acceptable range. However, repositioning the membranous septum and employing balloon post-dilation can diminish the incidence of atrioventricular block. In this case, we performed repositioning of the membranous septum using Ueki et al.’s technique [10] and controlled balloon post-dilation to prevent conduction disturbances. In our experience, no conduction disturbances emerged, demonstrating that sutureless AVR can be performed safely even when emergent revisions, such as a myectomy, are required immediately following valve implantation.

## Conclusions

We report a case in which SAM developed following aortic declamping, necessitating an additional myectomy. While SAM was not fully anticipated preoperatively, it is a recognized risk following mitral valve repair due to a combination of anatomical and hemodynamic factors. The use of a sutureless Perceval valve enabled rapid explantation and reimplantation, effectively minimizing prolonged myocardial ischemia. In addition to reducing ischemic time, the use of a sutureless valve contributed to surgical efficiency by simplifying the procedure and facilitating prompt intraoperative management. This case supports the role of sutureless valves particularly in scenarios requiring rapid intraoperative revision, such as SAM with LVOT obstruction, underscoring their value in managing unexpected yet recognized complications during complex cardiac procedures.
